# [Retracted] Synergistic inhibition of leukemia WEHI-3 cell growth by arsenic trioxide and *Hedyotis diffusa* Willd extract *in vitro* and *in vivo*

**DOI:** 10.3892/etm.2026.13175

**Published:** 2026-04-29

**Authors:** Yu-Jui Kuo, Yan-Jin Liu, Tzong-Der Way, Su-Yin Chiang, Jaung-Geng Lin, Jing-Gung Chung

Exp Ther Med 13:3388-3396, 2017; DOI: 10.3892/etm.2017.4392

Following the publication of the above paper, it was drawn to the Editor’s attention by a concerned reader that western blot data shown in Fig. 6A on p. 3394 were strikingly similar to data that had already appeared previously in an article written by different authors at different research institutes in the journal *PLoS One*. After performing an independent analysis of the data in this paper in the Editorial Office, it came to light that western blot data in Fig. 5C had also similarly appeared in the journal *Cancer Research* in a paper written by different authors at different research institutes.

In view of the fact that the abovementioned data had already apparently been published previously, the Editor of *Experimental and Therapeutic Medicine* has decided that this paper should be retracted from the Journal. The authors were asked for an explanation to account for these concerns, but the Editorial Office did not receive a reply. The Editor apologizes to the readership for any inconvenience caused.

